# Non-coding RNAs as potential biomarkers and therapeutic targets in polycystic kidney disease

**DOI:** 10.3389/fphys.2022.1006427

**Published:** 2022-09-20

**Authors:** Qi Zheng, Glen Reid, Michael R. Eccles, Cherie Stayner

**Affiliations:** Department of Pathology, Dunedin School of Medicine, University of Otago, Dunedin, New Zealand

**Keywords:** non-coding RNAs, miRNAs, long non-coding RNAs, polycystic kidney disease, autosomal dominant polycystic kidney disease

## Abstract

Polycystic kidney disease (PKD) is a significant cause of end-stage kidney failure and there are few effective drugs for treating this inherited condition. Numerous aberrantly expressed non-coding RNAs (ncRNAs), particularly microRNAs (miRNAs), may contribute to PKD pathogenesis by participating in multiple intracellular and intercellular functions through post-transcriptional regulation of protein-encoding genes. Insights into the mechanisms of miRNAs and other ncRNAs in the development of PKD may provide novel therapeutic strategies. In this review, we discuss the current knowledge about the roles of dysregulated miRNAs and other ncRNAs in PKD. These roles involve multiple aspects of cellular function including mitochondrial metabolism, proliferation, cell death, fibrosis and cell-to-cell communication. We also summarize the potential application of miRNAs as biomarkers or therapeutic targets in PKD, and briefly describe strategies to overcome the challenges of delivering RNA to the kidney, providing a better understanding of the fundamental advances in utilizing miRNAs and other non-coding RNAs to treat PKD.

## 1 Introduction

Polycystic kidney disease (PKD) is an inherited disorder in which fluid-filled cysts enlarge the kidney, resulting in kidney failure. Autosomal dominant polycystic kidney disease (ADPKD) and autosomal recessive polycystic kidney disease (ARPKD) are the two main types of PKD, with the more common adult form ADPKD having an estimated prevalence of between 1:1,000 and 1:2,500 (Torres et al., 2007; Willey et al., 2017). *PKD1* mutations are responsible for 80% of ADPKD cases, while around 15% of cases are attributed to *PKD2* mutations. The remaining cases are due to rare or unknown genetic abnormalities in other loci (Vasileva et al., 2021). ARPKD is less common than ADPKD with a reported incidence of 1 in 26,500 live births (Bergmann et al., 2018). ARPKD usually develops perinatally or in childhood, and most commonly results from missense or truncating mutations in *PKHD1*. The vasopressin 2 receptor antagonist tolvaptan is the only Food and Drug Administration (FDA)-approved drug for use in ADPKD, and can delay the progression of ADPKD but exhibits side effects like polyuria and liver damage (Muller et al., 2021). No drug has been approved for ARPKD treatment, however several potential drugs for ADPKD have been in clinical trials.

In the last 2 decades, increasing evidence supports key roles for non-coding RNAs (ncRNAs) in ADPKD pathogenesis. NcRNAs are classified into long ncRNAs (lncRNAs) and small ncRNAs, based on a size cut-off of 200 nucleotides. LncRNAs can be further divided into two types, linear lncRNAs and circular RNAs (circRNAs), and are characterized by the absence of a long open reading frame. Small ncRNAs comprise microRNA (miRNA), small interfering RNA (siRNA), piwi-interacting RNA (piRNA), transfer RNA-derived stress-induced small siRNA (tiRNA), small nuclear ribonucleic acid (snRNA), small nucleolar RNA (snoRNA), repeat-associated small interfering RNA (rasiRNA), small cajal body-specific RNA (scaRNA) and others (Zhou and Li, 2021). Among them, miRNAs are small single strand non-coding RNAs containing 19–25 nucleotides, of which a seed sequence, usually 2 to 8 bases, can perfectly or imperfectly bind to the 3’ untranslated region of complementary target mRNAs to mediate gene expression by translational repression or mRNA cleavage. MiRNAs are the most extensively investigated ncRNAs in a variety of diseases such as cancer, diabetes and kidney diseases, and their therapeutic application is the nearest to clinical development. Recent evidence has shown that ncRNAs, especially miRNAs, regulate cyst growth through diverse mechanisms, and could serve as potential biomarkers and therapeutic targets or tools. Thus, this review focuses on the current understanding of the molecular mechanisms by which ncRNAs and especially miRNAs contribute to the pathogenesis of PKD. We discuss advances in the understanding of ncRNAs as biomarkers as well as therapeutic targets or tools in PKD, and methods for delivery of these compounds to the kidney.

## 2 MicroRNAs in polycystic kidney disease pathogenesis

MiRNAs have been found to be expressed aberrantly in PKD patients and also in multiple PKD models by miRNA microarray, miRNA sequencing or RT-qPCR (Choi et al., 2021) (Dweep et al., 2013). Individual members of the miR-17–92 cluster represent differentially expressed miRNAs (DE-miRNAs) in both human and mouse ADPKD kidneys. Overexpression of the miR-17–92 cluster promoted tubular cysts and glomerular cysts by both directly targeting *Pkd1* and *Pkd2* via miR-17-5p and indirectly through inhibiting *Pkd2* and *Pkhd1* by miR-92a-3p targeting the transcription factor hepatocyte nuclear factor-1β (*Hnf-1β*) (Hajarnis et al., 2015).

Several aberrantly expressed factors have been shown to contribute to DE-miRNAs in PKD. Inactivation of Dicer*,* which is a necessary RNase III endonuclease that processes pre-miRNAs into mature miRNAs, resulted in downregulation of the miR-200 cluster and formation of tubular and glomerular cysts (Patel et al., 2012). RNA helicase p68, which forms a key ternary complex with Drosha for miRNA biogenesis, was elevated in ADPKD and its knockdown reduced levels of mature miR-17-5p, miR-200c-3p, and miR-182-5p. On the other hand, changes in regulation of miRNAs by transcription factors overexpressed in PKD has also been reported, including HNF-1β regulating the miR-200 cluster (Hajarnis et al., 2015)*,* c-Myc regulating miR-17-5p (Hajarnis et al., 2017), and CREB regulating miR-21-5p (Lakhia et al., 2016). The proposed roles of significant DE-miRNAs in PKD are discussed below and summarized in Table 1.

**TABLE 1:** A summary of published dysregulated miRNAs in Polycystic Kidney Disease.

microRNA	Up or down regulated	Methods used	Species/model	Experimentally validated gene target	Mechanism	References
miR-132-3p	up	miRNA Microarray, qPCR	*Pkd1* ^*F/F*^ *:HoxB7-cre* mouse; human ADPKD kidney tissue; human cystic cell line WT9-12; PKD/Mhm heterozygous (cy/+) rats	*FOXO3*	oxidative stress	Choi et al. (2021); Dweep et al. (2013)
miR-146b-5p	up	miRNA Microarray, qPCR	PKD/Mhm heterozygous (cy/+) rats	N/A	N/A	Dweep et al. (2013)
miR-1587-5p, and miR-3911-5p	down	qPCR	human ADPKD serum	N/A	N/A	Kocyigit et al. (2017)
miR-16-5p	up	qPCR	human ADPKD plasma	N/A	N/A	Kulesza et al. (2021)
miR-17–92 family	up	miRNA Microarray, *in situ* hybridization	human ADPKD kidney tissue; *Ksp/Cre; Pkd1* ^*F/F*^ mouse, *Ksp/cre;Kif3a* ^*F/F*^ mouse, *Pkhd1/cre;Pkd2* ^*F/F*^ mouse, *Pkhd1* ^−/−^ mouse, *Pkhd1/cre;Hnf-1β* ^*F/F*^ mouse	*Pparα, Pkd1, Pkd2, Hnf-1β*	mitochondrial metabolism, proliferation	Hajarnis et al. (2017); Patel et al. (2013); Yheskel et al. (2019); Lakhia et al. (2022)
miR-182-5p	up	miRNA Microarray, qPCR, miRNA-seq	ADPKD cyst-lined epithelial cells; *Pkd1* ^−/−^ mouse, *Pkd1* ^*F/F*^ *:HoxB7-cre* mouse	*Wasf2*, *Dock1*, and *Itga4*	fibrosis via TGF-β1/Smad3 signaling pathway, cytoskeleton rearrangement	Sun et al. (2020); Woo et al. (2017)
miR-192-5p, miR-194-5p	down	qPCR, *in situ* hybridization, small RNA-seq	human ADPKD kidney tissue, human ADPKD urinary exosomes; *Pkd1* ^*F/F*^ *:HoxB7-Cre* mouse	*ZEB2, CDH2*	EMT	Kim et al. (2019); Magayr et al. (2020)
miR-193b-3p	down	miRNA Microarray, qPCR	human cystic cell line OX161, OX938, SKI-001, and SKI-002	*ErbB4*	proliferation	Streets et al. (2017)
miR-199a-5p	up	miRNA Microarray, qPCR	human ADPKD kidney tissue; Han: SPRD cystic rats, PKD/Mhm heterozygous (cy/+) rats; human cystic cell line OX161 and WT9-12	*CDKN1C/p57*	apoptosis	Sun et al. (2015); Dweep et al. (2013)
miR-200 cluster (miR-200a-3p, miR-200b-3p, and miR-429-3p)	down	qPCR	Mouse renal epithelial cells (53A cells) that express dominant-negative mutant *Hnf-1β* (DN-*Hnf1β*); *Hnf-1β* mutant mouse	N/A	N/A	Hajarnis et al. (2015)
miR-200b-3p, miR-200c-3p, and miR-429-3p	up	qPCR	exosomes from mouse PH2 cells and *Pkd1* null mouse PN24 cells; *Pkd1* ^*RC/RC*^ mouse, *Pkd1* ^*flox/flox*^ *:Pkhd1-Cre* mouse	N/A	proliferation	Ding et al. (2021)
miR-21-5p	up	miRNA Microarray, qPCR	human ADPKD kidney tissue, human ADPKD serum; *Ksp/Cre; Kif3a* ^*F/F*^ mouse*, Pkhd1/Cre; Pkd2* ^*F/F*^ mouse, *Ksp/Cre; Pkd1* ^*F/F*^ mouse, *Ksp/Cre; Hnf-1β* ^*F/F*^ mouse; PKD/Mhm heterozygous (cy/+) rats	*Pdcd4*	apoptosis	Lakhia et al. (2016); Dweep et al. (2013); Kocyigit et al. (2017)
miR-214-3p	up	miRNA Microarray, qPCR, and *in situ* hybridization	*Pkhd1/Cre; Pkd2* ^*fl/fl*^ mouse, *Ksp/Cre; Pkd1* ^*fl/fl*^ mouse, PKD/Mhm heterozygous (cy/+) rats	*Tlr4*	inflammation	Lakhia et al. (2020); Dweep et al. (2013)
miR-25-3p	up	qPCR	human ADPKD serum; *Pkd1* ^*flox/-*^ *;Ksp-Cre* mouse	*ATG14*	autophagy	Liu et al. (2020a); Kocyigit et al. (2017)
miR-29b-5p, miR-106a-5p	down	miRNA seq	*Pkd2* ^*F/F*^ *:HoxB7-Cre* mouse, *Pkd2* ^*−/−*^ mouse; human cystic cell line WT9–7 and WT9-12	*KLF12*	proliferation	Shin et al. (2018)
miR-30a-5p, and miR-30e-5p	down	small RNA-seq	human ADPKD urinary exosomes	N/A	N/A	Magayr et al. (2020)
miR-30d-5p	down	small RNA-seq, miRNA PCR Array	human ADPKD urinary exosomes; peri-cystic local microenvironment of *mcwPkd1* ^*(nl/nl)*^ mouse	N/A	N/A	Magayr et al. (2020); Patil et al. (2018)
miR-378a-3p, and miR-344f-5p	down	miRNA PCR Array	peri-cystic local microenvironment of *mcwPkd1* ^*(nl/nl)*^ mouse	N/A	N/A	Patil et al. (2018)
miR-3907-3p, and miR-92a-3p	up	qPCR	human ADPKD serum	N/A	N/A	Kocyigit et al. (2017)
miR-501-5p	up	miRNA Microarray, qPCR	human ADPKD kidney tissue; human cystic cell line WT9–7 and WT9-12	N/A	p53 proteasome degradation through the activation of the mTOR/MDM2 pathway	de Stephanis et al. (2018)
miR-503-5p, and miR-34a-5p	up	miRNA Microarray	PKD/Mhm heterozygous (cy/+) rats	N/A	N/A	Dweep et al. (2013)
miR-667-3p, miR-3074-5p, and miR-7b-3p	up	miRNA PCR Array	peri-cystic local microenvironment of *mcwPkd1* ^*(nl/nl)*^ mouse	N/A	N/A	Patil et al. (2018)

Abbreviations: EMT, epithelial-mesenchymal transition; qPCR, quantitative reverse transcription polymerase chain reaction; miR, microRNA gene; N/A, not available; small RNA-Seq, small RNA sequencing.

### 2.1 MicroRNAs modulate oxidative stress in mitochondrial metabolism

Although *Pkd1* and *Pkd2* are direct miR-17-5p targets, miR-17-5p upregulated by c-Myc was also able to enhance proliferation in cystic kidneys from *Pkd1* knockout (KO) ADPKD models, at least in part, by inhibiting mitochondrial oxidative phosphorylation through repressing *Pparα* (Hajarnis et al., 2017). Increased miR-132-3p in ADPKD was shown to increase mitochondrial superoxide levels by directly repressing its target gene *Foxo3a*. This transcription factor regulates the expression of *Gatm* to induce reactive oxygen species accumulation in ADPKD (Ishimoto et al., 2017), indicating that oxidative stress might be one of the key factors in the pathogenesis of ADPKD.

### 2.2 MicroRNAs dysregulate cell proliferation

Like development of tumour cells, cyst progression also requires sustained proliferative signaling (Lantinga-van Leeuwen et al., 2007). Over-activation of mTOR-regulated pathways is one of the critical pathogenic processes for hyper-proliferation in PKD (Ibraghimov-Beskrovnaya and Natoli, 2011). Upregulation of miR-501-5p in ADPKD cells and tissues activates mTOR by repressing *PTEN* and *TSC1,* resulting in cell proliferation (De Stephanis et al., 2018). Though mTOR is a key pathway for cyst growth, mTOR inhibitors are not recommended for treating ADPKD because of various side effects noted in clinical trials, including interfering with normal recovery from injury (Torres et al., 2010). Nevertheless, an mTOR inhibitor used at low doses or in combination with other effective drugs could be further investigated.

Normal cells have many genes that strictly control proliferation, but in tumours, as well as in cysts, these genetic suppressors are often inactivated, leading to accelerated cell proliferation. Downregulation of several miRNAs targeting classical proliferative signaling pathways such as EGFR/ErbB1 has been demonstrated in association with ADPKD (Harskamp et al., 2016). MiR-193a-3p which contributes as a tumor suppressor miRNA in many cancer types (Khordadmehr et al., 2019) was reported to be downregulated in cystic cell lines derived from ADPKD human kidneys. Reduced miR-193a-3p led to increased expression of its target, the EGF/ErbB family receptor *ErbB4,* in human and mouse ADPKD cysts, resulting in cyst proliferation (Streets et al., 2017).

### 2.3 MicroRNAs respond to cell damage

Cellular mechanisms that protect against cell damage, such as autophagy and apoptosis, also play a distinct role in cell survival in PKD. Genes positively regulating autophagy were highly expressed in ADPKD patients samples in an online public dataset (GSE35831) (Liu et al., 2019), and inhibition of an autophagy suppressor in cysts indicated that autophagy is a key component in the development of PKD. MiR-25-3p was reported to be increased in *Pkd1*-KO mice. Inhibition of miR-25-3p augmented autophagy, while silencing its target, *ATG14,* abolished the effect of miR-25-3p inhibition in a model of PKD (Liu et al., 2020).

Apoptosis is another crucial mechanism in PKD where miRNAs participate. For example, miR-199a-5p has been reported to be highly expressed, both in human ADPKD tissues and in an ADPKD rat model. Antisense inhibition of miR-199a-5p induced apoptosis, which was suggested by the authors as likely due to the action of its target gene, *CDKN1C/p57*, an important cell cycle regulator (Sun et al., 2015). Additionally, increased expression of a conserved onco-microRNA, miR-21-5p, has been found in cyst epithelial cells from various PKD mouse models and human ADPKD patients (Lakhia et al., 2016). Specifically, miR-21-5p was shown to be associated with cyst expansion through apoptosis suppression, rather than cyst initiation.

### 2.4 MicroRNAs play a role in inflammation

Proinflammatory cytokines such as IL-1β, TNF-alpha, and IL-2 are present in the cyst microenvironment of human ADPKD kidneys (Gardner et al., 1991), and macrophages accumulate in cystic areas of the kidney (Karihaloo et al., 2011). High expression of interstitial miR-214-3p and its host lncRNA *Dnm3os,* derived from stromal cells rather than cyst epithelial cells, were validated by both *in situ* hybridization and single-cell RNA-Seq (Lakhia et al., 2020). Interestingly, this study also revealed that the proinflammatory TLR4/IFN-γ/STAT1 pathway activated miR-214-3p transcription and in turn, miR-214-3p directly inhibited *Tlr4*. Deletion of miR-214-3p resulted in elevated *Tlr4* mRNA expression, peri-cystic macrophage accumulation, and aggravated cyst growth, consistent with previous reports that macrophages promote cyst growth in ADPKD (Lakhia et al., 2020). These findings suggest that miR-214-3p generates a compensatory protective response in the cyst environment that restrains cyst inflammation.

### 2.5 MicroRNAs participate in Fibrosis

PKD is invariably associated with not only the expansion of cysts, but also the excessive deposition of extracellular matrix (ECM), resulting in ongoing fibrosis and loss of renal function leading to end-stage kidney disease (Xue and Mei, 2019). In PKD, the mechanisms for kidney fibrosis include chronic inflammation, epithelial-mesenchymal transition (EMT), up-regulation of transforming growth factor-β1 (*TGF-β1*) and increased expression of interstitial ECM proteins (Xue and Mei, 2019).

Previous profiling of end-stage ADPKD kidneys has shown increased expression of EMT-associated genes. Additionally, cystic epithelial cells express mesenchymal markers in the genotypic ADPKD Pck rat model, supporting a role for EMT in the pathogenesis of PKD (Norman, 2011). MiR-192-5p and -194-5p are both enriched in normal kidney but were found at decreased levels in the cysts of end-stage ADPKD human and mouse tissues (Kim et al., 2019). When miR-192-5p and -194-5p precursors were introduced into cysts, they were found to retard cyst growth by reducing expression of the EMT-associated genes *ZEB2* and *CDH2*. Another miRNA, miR-182-5p, was also positively correlated with fibrosis and elevated the expression of collagen I, collagen IV and fibronectin in cystic epithelial cells (Sun et al., 2020). These findings indicate that dysfunctional miRNA expression in cystic epithelial cells contributes to fibrosis and might represent novel prognostic biomarkers or therapeutic targets for the progression of PKD.

The sources of ECM production in ADPKD are likely to be a heterogeneous population and are not limited to cystic epithelial cells. Myofibroblasts, which exhibit features of fibroblasts and smooth muscle cells, are generally considered to be the major source of ECM, leading to renal scarring and fibrosis. Transition of different cell types into activated interstitial myofibroblasts might also occur during renal fibrosis (Meng et al., 2016). In pericyte-derived myofibroblasts, silencing miR-132 via *TGF-β* signaling (*Smad2/Smad3*) attenuated the progression of renal fibrosis by selectively decreasing myofibroblast proliferation (Bijkerk et al., 2016). Crosstalk signaling, including via miRNAs within diverse cells types, potentially participates in the progression of cystic epithelial cells and accumulated fibrosis.

### 2.6 MicroRNAs are associated with cell-to-cell contacts via extracellular vesicles and exosomes

Bioinformatic analysis of two public GEO datasets (GSE7869 and GSE35831) of human ADPKD revealed that differentially expressed genes were enriched in extracellular exosomes (Liu et al., 2019). Cystic cell derived extracellular vesicles (EVs) and urinary exosomes derived from ADPKD patients promoted cyst growth in *Pkd1* mutant kidneys and in 3D cultures (Ding et al., 2021). This function was partially achieved by microRNAs which are a part of the diverse array of biomolecules contained within EVs/exosomes. Increased levels of miR-200b-3p, miR-200c-3p, and miR-429-3p were secreted in EVs/exosomes derived from *Pkd1*-null renal epithelial cells (Ding et al., 2021). Inhibition of exosome biogenesis/release with GW4869 significantly delayed cyst growth in both aggressive and milder ADPKD mouse models (Ding et al., 2021). These findings suggest that EVs/exosome-derived biomolecules (including miRNAs) crucially influence cell-to-cell contacts. Therefore, exosomes and their miRNA cargoes have both diagnostic and therapeutic potential for ADPKD, and their significance in PKD will likely expand in the future.

## 3 LncRNAs and polycystic kidney disease

LncRNAs have diverse functions, including as guides, scaffolds, or activators for transcription factors and histone modifiers. They influence posttranscriptional regulation by affecting mRNA activity and stability, as well as translational efficiency. LncRNAs have been implicated in a range of diseases including various types of cancer, and recently their involvement in ADPKD has also been reported. *Makorin1-p1* is a lncRNA, downregulation of which induced polycystic kidney and bone deformity in transgene-insertion-induced *Makorin1-p1* mutant mice (Ivanova et al., 2003). This lncRNA shares a homologous 5’ region with the protein-encoding gene *Makorin-1* and potentially protects this mRNA by competitive binding to a trans-acting RNA-destabilizing factor, whose binding site is within the homologous region of *Makorin-1*, increasing *Makorin-1* mRNA stability (Ivanova et al., 2003). As mentioned previously, *Dnm3os,* the host lncRNA of miR-214-3p, was upregulated in orthologous ADPKD mouse models and cystic kidneys from ADPKD patients (Lakhia et al., 2020). Although its function has not been studied further in a PKD context, overexpression of *Dnm3os* in macrophages alters global histone modification and enhances inflammation (Das et al., 2018), indicating a possible role for *Dnm3os* in ADPKD. Similarly, HNF-1β, which directly regulates *Pkd2* and *Pkhd1,* also binds to the promoter of a 28 kb lncRNA that encodes the miR-200b-3p/200a-3p/429-3p cluster, to regulate the expression of *Zeb2* and *Pkd1* (two target genes of miR-200b-3p), indirectly producing cysts (Hajarnis et al., 2015). RNA-seq has identified a kidney-specific, evolutionarily conversed lncRNA called *Hoxb3os* that was highly expressed in renal tubules in adult wild-type mice but down-regulated in cystic kidneys from *Pkd1* and *Pkd2* mutant mice. The human ortholog *HOXB3-AS1* was also down-regulated in cystic kidneys from ADPKD patients (Aboudehen et al., 2018). Knock-out of *Hoxb3os* increased phosphorylation of mTOR and its down-stream targets, namely p70 S6 kinase, ribosomal protein S6, and the translation repressor 4E-BP1. Through activating mTOR signaling, *Hoxb3os*-KO cells displayed increased mitochondrial respiration. Exactly how *Hoxb3os* regulates the mTOR signaling is still unclear, but these findings indicate functional roles for lncRNAs in PKD.

In addition, lncRNAs that mediate expression of *PKD1* in other diseases might be implicated in PKD and require further investigation. For example, lncRNA H19 can recruit CCCTC-binding factor to the promoter of *PKD1*, and inhibit the transcription of *PKD1* in atherosclerotic vulnerable plaques (Yang et al., 2019). Furthermore, through bioinformatic analysis the *PKD1–*miR-20b-5p–*AP000797* axis was predicted to regulate intervertebral disc degeneration (Cao et al., 2021).

## 4 Prospective applications of non-coding RNAs in polycystic kidney disease

### 4.1 Emerging biomarkers as liquid biopsy

Due to the relative stability of cell-free circulating miRNAs (as well as miRNAs and lncRNAs found in exosomes) in multiple body fluids, ncRNAs (and especially miRNAs) have become candidate biomarkers in liquid biopsy for various diseases. Compared with healthy subjects, serum from ADPKD patients contained increased levels of miR-3907-3p, miR-92a-3p, miR-25-3p and miR-21-5p, and decreased levels of miR-1587-5p and miR-3911-5p as renal function declined. Among these, an increasing level of miR-3907-3p had a potential predictive value for disease progression (Kocyigit et al., 2017). Profiling revealed that another four miRNAs are dysregulated in ADPKD urine specimens, compared to specimens of other chronic kidney diseases (Ben-Dov et al., 2014). In an initial discovery cohort of ADPKD patients with early or late disease, compared with healthy controls, profiling of human urinary exosome miRNA demonstrated that miR-192-5p, miR-194-5p, miR-30a-5p, miR-30d-5p and miR-30e-5p were significantly downregulated in patient urine exosomes, in both human *PKD1* cystic kidney tissue, and in murine *Pkd1* cystic kidneys. All five miRNAs showed significant correlation with baseline eGFR and ultrasound-determined mean kidney length, and improved the diagnostic performance (area under the curve) of mean kidney length for the rate of disease progression (Magayr et al., 2020).

In addition to biomarkers for disease progression, miRNAs can also serve as predictive factors for complications of ADPKD. For example, plasma levels of miR-16-5p were reported to be a predictive factor for intracranial aneurysms in renal transplant recipients with ADPKD, and may become a tool to identify high-risk groups who should undergo magnetic resonance angiography for intracranial aneurysm screening (Kulesza et al., 2021).

### 4.2 Potential therapeutic targets for polycystic kidney disease

The only ncRNA-based therapeutic approach for ADPKD to reach clinical trial to date is the use of antisense oligonucleotides to inhibit miR-17-5p. Preclinical results showed that the anti-miR-17-5p oligonucleotide RGLS4326 attenuated cyst growth in multiple PKD models (Lee et al., 2019). Interestingly, a recent publication demonstrated de-repression of *Pkd1* and *Pkd2* as a result of CRISPR/Cas9-mediated deletion of 3’-UTR miR-17-5p binding sites. This significantly alleviated cyst growth with decreased kidney-weight-to-body-weight ratio, decreased serum blood urea nitrogen levels, improved mitochondrial membrane potential, and inhibition of the pro-cystogenic effect of cAMP. These results were observed in cellular, *ex vivo*, and mouse ADPKD models (Lakhia et al., 2022). Moreover, blockade by RGLS4326 of *Pkd1/2* cis-inhibition prevented cyst onset and stabilized existing cysts in mice (Lakhia et al., 2022). However, dose-limiting central nervous system toxicity was observed in both mice and monkeys receiving high doses of RGLS4326 due to the off-target inhibition of the neuroreceptor α-amino-3-hydroxy-5-methyl-4-isoxazolepropionic acid receptor (AMPA-R) by RGLS4326 (Edmund Lee, 2022). To overcome this toxicity, a next-generation anti-miR-17-5p oligonucleotide RGLS8429 with similar efficacy as RGLS4326, but no affinity to AMPA-R, has been developed and is currently being evaluated in a Phase 1 clinical trial (NCT05429073).

To complement sequence-based recognition of oligonucleotides, structure-binding small molecules also have huge potential for clinical application. Primary miRNAs and lncRNAs have specific hairpin structures which are favorable regions for structure-specific ligands to target. A ligand that targets the primary miR-17-92 cluster reduced its expression in PKD, prostate cancer, and breast cancer and was shown to rescue disease-associated phenotypes in the latter two (Liu et al., 2020b). Application of this ligand and its chemical modification, or optimized conjugation to an endogenous nuclease or a ribonuclease targeting chimera as an RNA-degradation targeting treatment for PKD warrants further investigation.

## 5 Delivery of non-coding RNAs to the kidney

**FIGURE 1:**
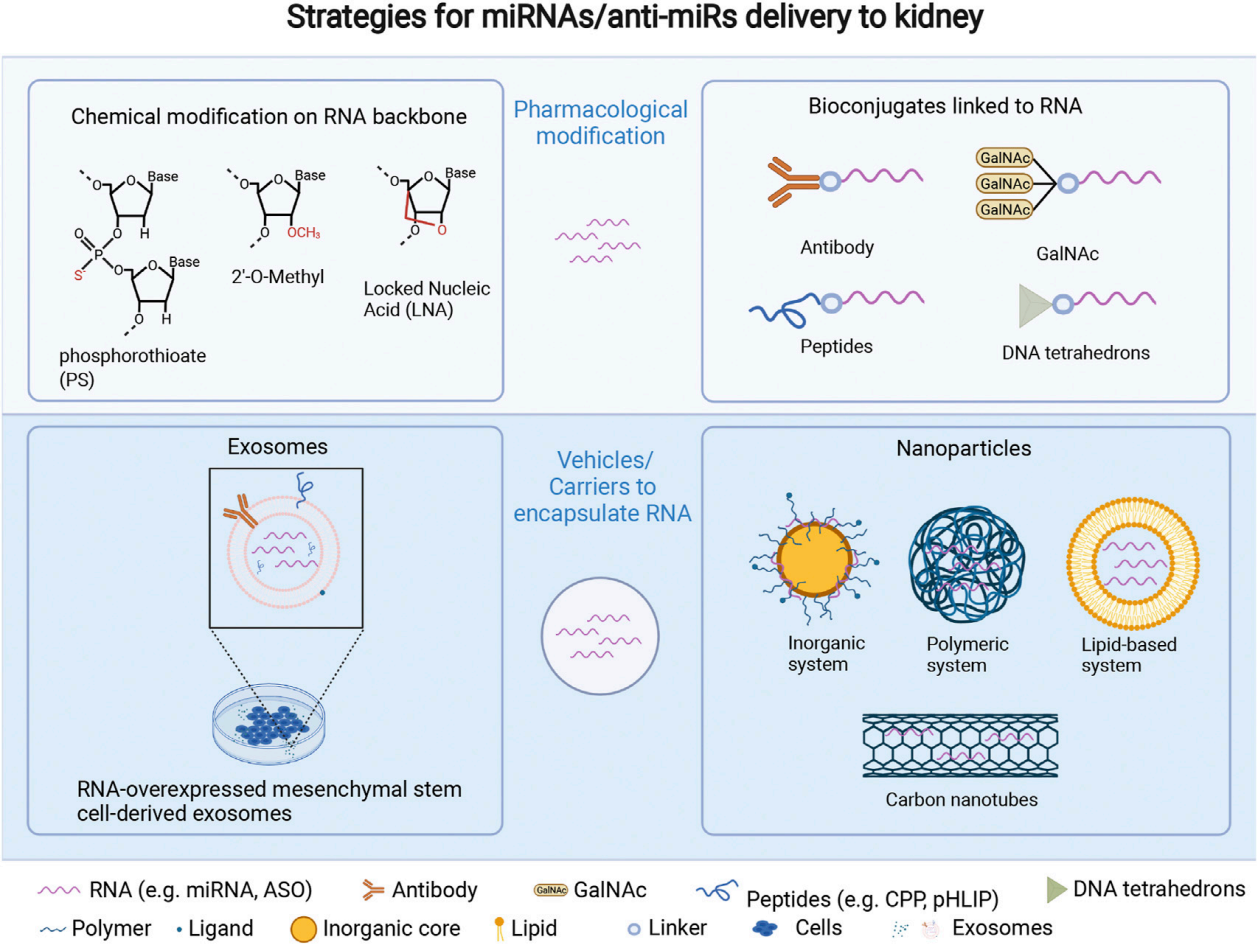
Strategies for miRNA/anti-miR delivery to kidney, which include pharmacological modification of miRNAs/anti-miRs, and vehicles/carriers embedding miRNAs/anti-miRs. Pharmacological modification: 1) chemical modifications such as phosphorothioate (PS) (Yheskel et al., 2019), 2ʹ-O-methyl (Straarup et al., 2010), and locked nucleic acid (Lendvai et al., 2005) all result in a high kidney uptake through intravenous injection. 2) Bioconjugates can address some oligonucleotide delivery challenges, such as improving biodistribution to a specific region or cell type (e.g., antibodies, DNA tetrahedrons) (Benizri et al., 2019), promoting endosomal escape (e.g., CPP) (Miguel et al., 2020), increasing receptor-mediated transport (e.g., GalNAc), and/or improving ability to cross cell and endosomal membranes (e.g., pHLIP). (Rohde et al., 2015; Wagner, 2015). Delivery vehicles or carriers comprise two main methods. 1) exosomes derived from various cell types, like mesenchymal stem cells, have the potential to be drug carriers for ncRNAs or oligonucleotides due to their stability, minimal immune response, and editable surface with various RNAs loading strategies including diffusion via a concentration gradient, transfection, or physical treatments like electroporation, which can also expand the cargo loading capability of exosomes (Jin et al., 2021). 2) Nanoparticles, which refer to a stable structure with a protective layer that can encapsulate and protect inner agents, and be modified with ligands or antibodies on their surface for kidney-specific targeting at a nano scale, have the potential to improve the pharmacokinetics, biodistribution, toxicity, and efficacy of encapsulated drugs: 1) inorganic system like pSi and magnetic particles (Tsai et al., 2019); 2) polymeric system including synthetic polymerics like polymeric CXCR4 inhibitors (Tang et al., 2022) and natural polymers such as chitosan (Chen et al., 2019); 3) lipid-based system (Su et al., 2022); 4) carbon-nanotubes (Alidori et al., 2016). LNA = locked nucleic acid; CPP = cell penetrating peptide; GalNAc = N-acetylgalactosamine; pHLIP = pH (low) insertion peptides. Created with Biorender.com.

Although single-stranded, chemically modified, antisense molecules such as RGLS4326 can be administered without a carrier (Lee et al., 2019), replacing suppressed microRNAs (using double-stranded RNA precursors) is more difficult. Although few miRNA mimics or siRNA-based therapies in PKD have been studied, research on RNA delivery in other kidney diseases might be informative and can be divided into pharmacological modification or use of delivery vehicles or carriers (Figure 1). Pharmacological modification of RNAs is an effective way to overcome poor stability, immune responses and to change the tissue-specific uptake. Due to the limitations of viral vectors, non-viral vehicle platforms to deliver RNAs to kidney such as nanoparticles and engineered exosomes have been advanced to stabilize RNAs and overcome poor cellular uptake in kidney disease. Other novel technologies, such as combinations of focused ultrasound and gas-filled microbubbles (Wonnacott et al., 2022) or virus-like particles (Segel et al., 2021) might also be applied to kidney and PKD treatment in the near future. A hurdle may be non-specific liver accumulation, although systemically administered carrier-free short RNAs do appear to preferentially accumulate in the kidney (Denby et al., 2014; Lee et al., 2019). This is possibly due to their hydrophilic nature, size and negative charge which could affect glomerular filtration and absorption, and release into cyst-related cells such as cyst-lining tubular epithelial cells, and cyst-surrounding macrophages (Fernandez-Pineiro et al., 2017).

## 6 Conclusion

MiRNAs and lncRNAs are emerging as indispensable regulators in the development of PKD. The discovery of aberrantly expressed ncRNAs in both PKD models and patient samples, provide clues for new molecular mechanisms of PKD pathogenesis and potential applications as biomarkers or therapeutic targets. Nevertheless, there are significant gaps, such as observed differences in efficacy between PKD animal models and clinical trials, issues with kidney-specificity of ncRNA delivery, and also off-target effects observed between ncRNA preclinical studies and clinical trials for PKD. The preclinical and clinical outcomes of RGLS4326 and RGLS8429 may provide valuable knowledge for ncRNA-based therapeutics in PKD. Moreover, precise ncRNA profiling of human specimens are still challenging and require further optimization. Importantly, the potential roles of other ncRNAs (in addition to miRNAs and lncRNAs) in PKD remain uninvestigated. Further studies are needed to support the emerging implementation of ncRNAs as therapeutic biomarkers and targets for patients with PKD.
